# Disturbance history can increase functional stability in the face of both repeated disturbances of the same type and novel disturbances

**DOI:** 10.1038/s41598-020-68104-0

**Published:** 2020-07-09

**Authors:** Sophia Elise Renes, Johanna Sjöstedt, Ingo Fetzer, Silke Langenheder

**Affiliations:** 1 Department of Aquatic Sciences and Assessment, Swedish University of Agricultural Sciences, Uppsala, Sweden; 2 Department of Ecology and Genetics/Limnology, Uppsala University, Uppsala, Sweden; 3 Department of Biology/Aquatic Ecology, Lund University, Lund, Sweden; 4 Stockholm Resilience Centre, Stockholm University, Stockholm, Sweden; 5 Bolin Centre for Climate Research, Stockholm University, Stockholm, Sweden

**Keywords:** Microbial ecology, Microbial communities, Environmental microbiology

## Abstract

Climate change is expected to increase the incidences of extremes in environmental conditions. To investigate how repeated disturbances affect microbial ecosystem resistance, natural lake bacterioplankton communities were subjected to repeated temperature disturbances of two intensities (25 °C and 35 °C), and subsequently to an acidification event. We measured functional parameters (bacterial production, abundance, extracellular enzyme activities) and community composition parameters (richness, evenness, niche width) and found that, compared to undisturbed control communities, the 35 °C treatment was strongly affected in all parameters, while the 25 °C treatment did not significantly differ from the control. Interestingly, exposure to multiple temperature disturbances caused gradually increasing stability in the 35 °C treatment in some parameters, while others parameters showed the opposite, indicating that the choice of parameters can strongly affect the outcome of a study. The acidification event did not lead to stronger changes in community structure, but functional resistance of bacterial production towards acidification in the 35 °C treatments increased. This indicates that functional resistance in response to a novel disturbance can be increased by previous exposure to another disturbance, suggesting similarity in stress tolerance mechanisms for both disturbances. These results highlight the need for understanding function- and disturbance-specific responses, since general responses are likely to be unpredictable.

## Introduction

Microbial communities perform essential functions in different ecosystems, including decomposition, primary production and nitrogen fixation^1–3^. Since natural communities undergo regular disturbances, and the incidences of extremes in environmental conditions are expected to increase as climate change becomes more severe^4^, it is essential to investigate how more frequent pulse disturbances affect the ability of microbial communities to cope with a changing environment in order to maintain essential ecosystem services.

The effect of disturbances on the composition and function of communities can be addressed in terms of resistance, recovery and resilience, where resistance is defined as the insensitivity to a disturbance and recovery and resilience (more specifically engineering resilience) as the degree and rate of recovery after a disturbance^5^. Previous reviews of the literature have shown that microbial communities are mostly not resistant, instead they often change in composition and function in response to disturbances^5–8^. In contrast, recovery and resilience are still rarely studied, so it remains unclear under which circumstances and at which rates compositional recovery occurs in microbial communities^5,8^. Changes in community composition following a disturbance may also underlie changes in ecological function, although this relationship is not well understood^9^. Often, communities undergo changes in composition without concomitant functional changes being found, which might be related to high functional redundancy within bacterial communities^5^. Moreover, there is evidence that compositional and functional stability in microbial communities are not necessarily linked^10^.

Most previous studies only considered effects of single pulse (short term) or press (long term) disturbances^1,11,12^. In natural systems, however, microbial communities are often exposed to multiple pulse and press disturbances that can occur either simultaneously or sequentially and, moreover, differ in frequency and intensity. Both bacterial community composition and functioning have been shown to change gradually with increasing frequency and intensity of disturbances^13,14^. The effect of multiple disturbances on bacterial communities has, however, so far only been investigated in relatively few studies^15–19^.

Multiple disturbances can either be of the same or a different type than previous disturbances that communities have been exposed to. This can affect the resistance, recovery and resilience of the communities. Multiple disturbances can have non-additive effects on communities^20,21^ and the effect of one disturbance can mitigate the effect of another disturbance, resulting in increased compositional resistance and resilience of the community^20,22,23^. Alternatively, exposure to one disturbance can leave a community more sensitive to subsequent disturbances^22,24^.

Exposure to multiple disturbances can affect the compositional and/or functional stability of microbial communities by different mechanisms. Firstly, exposure to previous disturbances can lead to a phenomenon called acquired stress resistance, where a first mild dose of one disturbance prepares cells to resist the second dose by activating different cellular mechanisms and minimizing metabolic costs^25–28^. In bacterial communities this physiological response may have long-lasting effects, resulting in changes in succession patterns^26^. A second mechanism explaining non-additive effects of multiple stressors is species co-tolerance^29^, where the impact of multiple stressors on a community is determined by the sign and magnitude of the correlation between species tolerance to two different disturbances. If this correlation is positive, then exposure to one stressor will pre-select for a community that is already more tolerant to a second stressor^29^. Conversely, if species tolerance to the stressors is negatively correlated, exposure to one stressor will result in a community that is particularly sensitive to the second stressor. These scenarios are named stress-induced community tolerance and stress-induced community sensitivity, respectively^29^. A possible explanation for the occurrence of stress-induced community tolerance is that disturbances can select for generalists, increasing their proportions in communities. This has been suggested based on experiments where bacterial communities exposed to disturbances showed enhanced physiological tolerance and substrate utilization^30,31^. Furthermore, exposure to reduced pH has been shown to induce a greater proportion of generalists in bacterial communities and result in a higher resistance to an additional salt disturbance^19^.

The aim of the present study was to investigate how exposure to repeated homogenous disturbances of different intensities affects the resistance and recovery of a microbial community, both in terms of composition and functioning, and how disturbance history affects resistance and recovery in response to a novel disturbance. We hypothesize that (1a) resistance and recovery of bacterial communities will change directionally (i.e. consistently either increase or decrease) over time when communities are exposed to recurring disturbances and that (1b) this effect will be stronger at higher disturbance intensities. Furthermore, we hypothesize that (2) having a history of frequent exposure to disturbances of one type, will modulate community responses to a novel type of disturbance. In order to test these hypotheses, natural lake bacterioplankton communities were subjected to weekly temperature pulse disturbances of two different intensities for 4 weeks, and subsequently exposed to an acidification disturbance.

## Results

### Experiment 1: Effect of repeated temperature disturbances at different intensities

In Experiment 1 we investigated how exposure to recurring temperature pulses affected the resistance and recovery of a microbial community and how this response was modulated by disturbance intensity.

### Community composition

**Figure 1:**
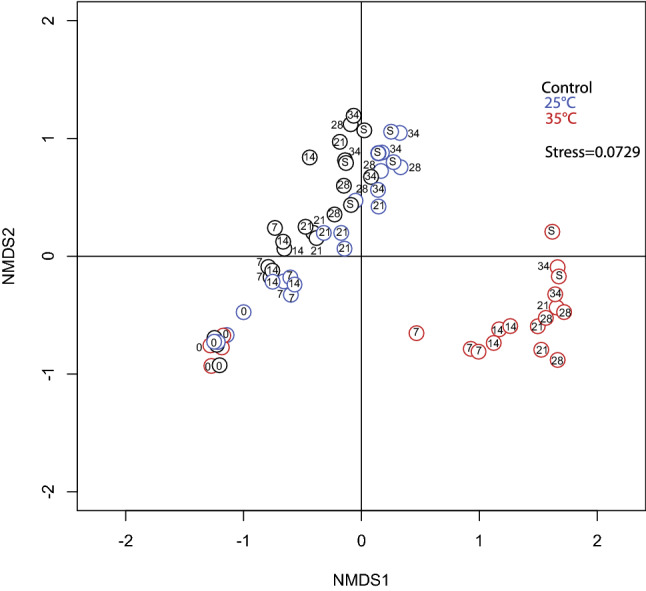
NMDS ordination plot based on 16S rRNA gene amplicon sequencing showing changes in bacterial community composition over time. Analysis is based on data from Illumina sequencing and Bray–Curtis dissimilarities. Numbers within circles denote the day of the experiment when the sample was taken and S denotes the samples exposed to pH disturbance (day 34). Treatment groups are indicated by the colour.

The NMDS plot based on sequence data distinguished 3 groups (Fig. 1): The first group contained all samples from day 0, the second group samples from the 35 °C treatment (days 7–28) and the third those from the control and 25 °C treatments (days 7–28). Measured as average Bray-Curtis dissimilarity, community composition changed most between days 0 and 7 (Supplementary Table S1) in all treatments. However, for the 25 °C treatments the change in community composition was almost as large between days 14 and 21 as between days 0 and 7 (Supplementary Table S1, 0.473 ± 0.108 respectively 0.528 ± 0.0377). The largest change was observed in the 35 °C treatments where the average Bray-Curtis dissimilarity was 0.910 ± 0.0156 between days 0 and 7.

**Figure 2:**
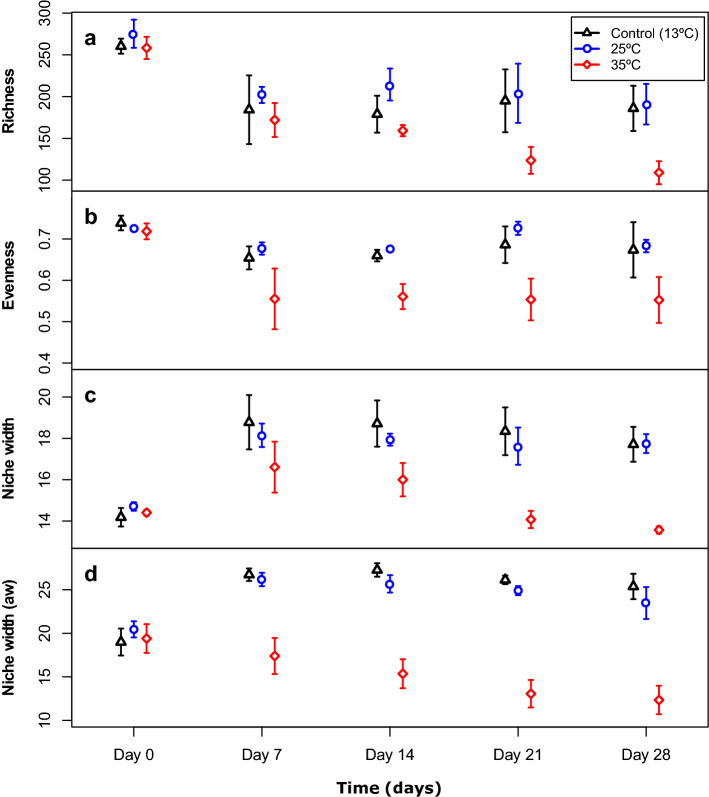
Changes in richness (**a**), evenness (**b**) and niche width (**c**,**d**) in Experiment 1 over time in the different treatments. Average niche width was calculated in two different ways, without (**c**) and with (**d**) taking the abundance of each OTU present in each sample into account (presence-absence versus abundance-weighted; aw). Points represent mean values (n = 4) and error bars indicate standard deviations.

Both temperature and time had highly significant effects on richness and evenness, whereas the interaction term was only marginally significant (Fig. 2, general linear mixed model ANOVA (mm ANOVA), Supplementary Table S2). For niche width (abundance-weighted and presence-absence), both main effects as well as the interaction terms were highly significant (Fig. 2, Supplementary Table S2).

Richness decreased between days 0 and 7 in all treatments. In the 35 °C treatment there was a continuous decrease, and richness was significantly lower than in the other two treatments on days 21 and 28 (Fig. 2, Supplementary Table S3). Evenness also decreased in the 35 °C treatment between days 0 and 7 and stayed at a similar level until the end of experiment 1. On day 7 evenness in the 35 °C treatment was significantly lower than in the 25 °C treatment, and on days 21 and 28 it was significantly lower compared to both other treatments (Fig. 2, Supplementary Table S3). Presence-absence based niche width first increased in all treatments, but subsequently decreased over time in the 35 °C treatment. From day 7 onward presence-absence based niche width was significantly lower in the 35 °C treatment than in the control, and from day 21 onward it was also significantly lower compared to the 25 °C treatment (Fig. 2, Supplementary Table S3). Abundance-weighted niche width showed a similar pattern, but in the 35 °C treatment it started to decrease immediately. Further, the difference between the 35 °C treatment and the other two treatments was already highly significant on day 7, and remained so until the end of Experiment 1.

### Community functioning

**Figure 3:**
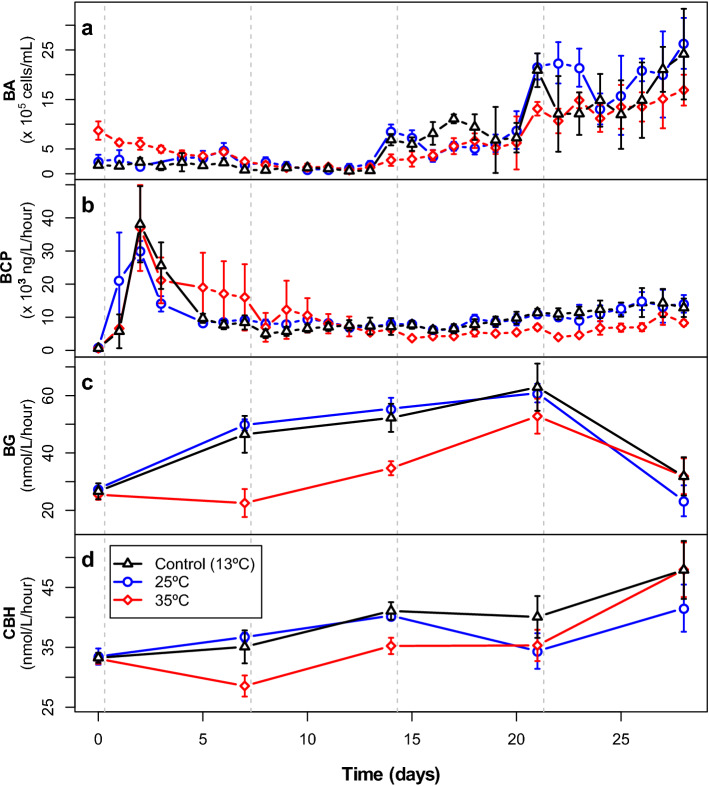
Bacterial abundance (BA; **a**), bacterial carbon production (BCP; **b**), β-glucosidase activity (BG; **c**) and cellobiohydrolase activity (CBH; **d**) in cultures during Experiment 1. Points represent mean values (n = 4) and error bars indicate standard deviations. Vertical dashed lines indicate the timing of the pulse disturbances.

**Figure 4:**
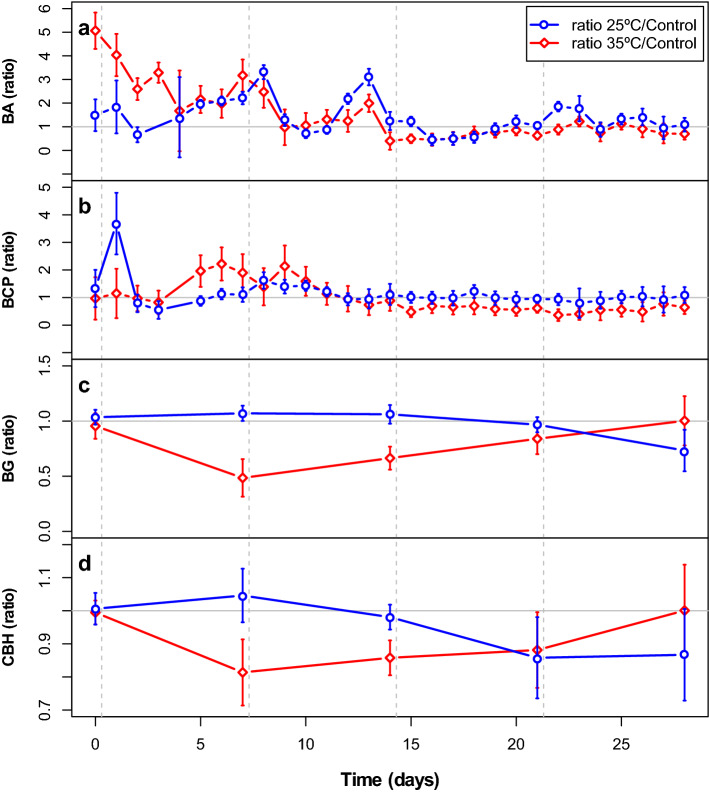
Response ratios in Experiment 1 based on bacterial abundance (BA; **a**), bacterial carbon production (BCP; **b**), β-glucosidase activity (BG; **c**) and cellobiohydrolase activity (CBH; **d**). Resistance was calculated as the ratio of the average bacterial abundance and bacterial production of the respective treatment and the control at each time point, respectively. Points represent mean values (n = 4) and error bars indicate standard deviations. Vertical dashed lines indicate the timing of the pulse disturbances, horizontal grey line indicates the 1:1 ratio.

Abundance and extracellular enzyme activities differed significantly between the temperature treatments, whereas bacterial carbon production did not (Fig. 3, multivariate repeated-measures ANOVA (rm ANOVA), Supplementary Table S4). In addition, all functional parameters changed significantly over time (Supplementary Table S4, Fig. 3) and the interaction between time and temperature treatment was significant as well. The first two temperature pulses were followed by strong changes in the response ratios of bacterial abundance (Fig. 4a). In the 25 °C treatment, response ratios of bacterial abundance decreased one or two days after the two first temperature disturbances, and then generally recovered within one or two days. However, bacterial abundances in the 25 °C treatment were most sensitive to the third disturbance where they reached the lowest value compared to the control (44%) and recovery occurred only after four days. In the 35 °C treatment the first two temperature pulses led to an increase in the response ratio of bacterial abundance and bacterial abundance remained higher than in the control (response ratios > 1) until day 9. On day 16, however, bacterial abundance decreased drastically and reached the lowest value compared to the control (40%) and the ratio remained below 1 for the rest of the experiment except for day 25 (Fig. 4a). Towards the end of the experiment the response ratios for the 25 °C treatment stabilized just above 1, whereas in the 35 °C treatment it stabilized just below 1 (Fig. 4a).

The first two disturbances also caused large fluctuations in the response ratios based on bacterial carbon production (Fig. 4b). After the first temperature pulse, the response ratios of bacterial carbon production decreased in both the 25 and 35 °C treatments. The lowest values were reached on day 3 in the 25 °C treatment (55% of the control) and day 4 (57% of the control) in the 35 °C treatment. Bacterial carbon production in the 25 °C treatment was less affected by the second, third and fourth temperature disturbances. In the 35 °C treatment, bacterial carbon production recovered after the first temperature disturbance and was unaffected or positively affected by the second temperature disturbance until day 12 (Fig. 4b). Toward the end of the experiment the response ratio for bacterial carbon production stabilized around 1 in the 25 °C treatment and around 0.8 in the 35 °C treatment (Fig. 4b).

The pattern for the response ratios of the enzyme activities was very different from those for bacterial abundance and carbon production. For both enzyme activities the response ratios in the 25 °C treatments were higher after the first two or three temperature pulses, compared to the later ones. Β-glucosidase activity was either resistant to the three first temperature disturbances or had (almost) fully recovered within seven days. After the fourth temperature pulse β-glucosidase activity was as low as 73% of the control (Fig. 4c). Similarly, cellobiohydrolase activity was resistant or resilient after the first two pulses, but then decrease to around 85% of the activity in the control after the third and fourth temperature pulses (Fig. 4d). Enzyme activity in the 35 °C treatment showed the opposite pattern, with a lower resistance to the first compared to the later disturbances. Specifically, β-glucosidase activity in the 35 °C treatment reached 48, 66 and 84% of the activity in the control treatment after the first, second and third disturbance, respectively, followed by full recovery after the last disturbance (Fig. 4c). Cellobiohydrolase activity decreased to between 80 and 90% of the controls after the three first disturbances, and recovered to the same activity level as the controls after the last disturbance (Fig. 4d).

### Experiment 2: Resistance to acidification in response to disturbance history

In Experiment 2, we investigated how the temperature disturbance history affected the response of the communities to a novel disturbance. Each microcosm from experiment 1 was split into two new ones, where one set was exposed to a pH disturbance and the other was used as a control.

### Community composition

One week after the acidification event (day 34), the samples from the control and the 25 °C treatments formed a group which was clearly separated from the samples from the 35 °C treatment. However, there was no clear pattern differentiating the pH disturbance from the pH control samples in any of the treatments (Fig. 1). The average Bray Curtis dissimilarities between day 28 and 34 did not differ between the control microcosms and the microcosms exposed to pH disturbance in the control treatments (0.384 ± 0.0981 respectively 0.366 ± 0.115). Whereas in the 25 °C and 35 °C treatments, the average Bray Curtis dissimilarity was slightly higher for the microcosms exposed to pH disturbance (0.317 ± 0.11 compared to 0.392 ± 0.0627 respectively 0.434 ± 0.078 compared to 0.548 ± 0.135).

### Community functioning

**Figure 5:**
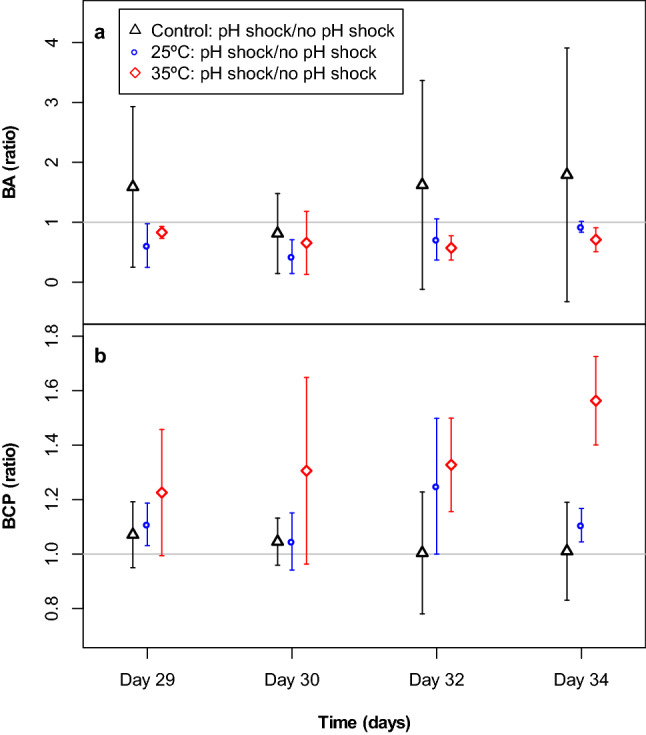
Response ratios in Experiment 2 based on bacterial abundance (BA; **a**) and bacterial carbon production (BCP; **b**). Points represent mean values, error bars indicate standard deviations. (n = 3 for the 35 °C treatment, all others: n = 4). Horizontal grey line indicates the 1:1 ratio.

Temperature disturbance history had a significant effect on the response ratio of bacterial abundance to the pH disturbance despite high variation among replicates (rm ANOVA *p* = 0.0083, Supplementary Tables S5 and S6). The average bacterial abundance in the pH treatments varied between 81% ± 67% and 179 ± 211% of the pH control in the temperature control treatment, between 42% ± 28% and 92 ± 9% of the pH control in the 25 °C treatment, and between 57% ± 20% and 83% ± 10% in the 35 °C treatment (Fig. 5a).

Temperature history also had a significant effect on the response ratios of bacterial carbon production (rm ANOVA *p* < 0.01, Supplementary Table S5). The average bacterial carbon production showed an upward trend and varied between 100 ± 22 and 107 ± 12% of the pH control in the temperature control treatment, between 104 ± 10% and 125 ± 25% of the pH control in the 25 °C treatment, and between 123 ± 23 and 156 ± 16% of the pH control in the 35 °C treatment (Fig. 5b). The response ratio for bacterial carbon production was significantly higher in the 35 °C treatment compared to the other two treatments on the last day of the experiment (Tukey’s HSD, *p* < 0.01 for both comparisons, day34, Supplementary Table S6).

**Figure 6:**
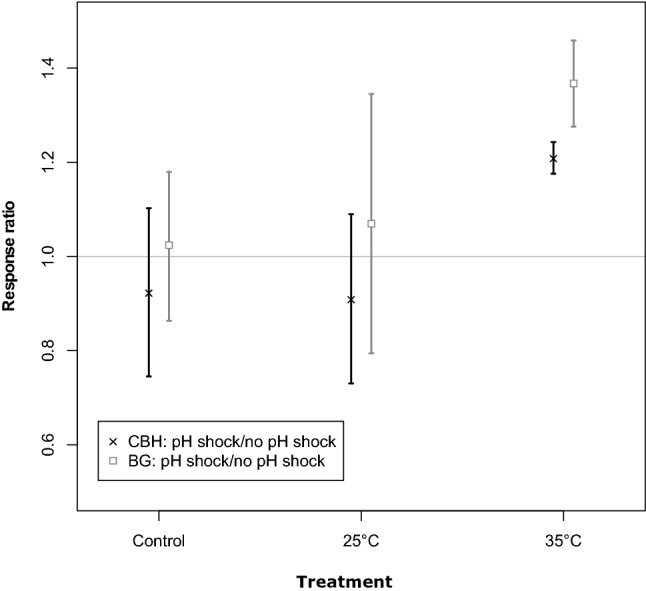
Response ratios in Experiment 2 based on enzyme activity: cellobiohydrolase (CBH) and β-glucosidase activity (BG). Enzyme activity was measured at day 34 and resistance was calculated as the ratio of the average functional parameter of respective treatment and the control at each time point. Points represent mean values, error bars indicate standard deviations. (n = 3 for the 35 °C treatment, all others: n = 4). Horizontal grey line indicates the 1:1 ratio.

Enzyme activities were only measured at the end of experiment 2, i.e. 1 week after the pH disturbance and no significant differences in response ratios were found between the temperature treatments (ANOVA, *p* = 0.0681 for cellobiohydroalse activity and *p* = 0.114 for β-glucosidase, Supplementary Table S6, Fig. 6).

## Discussion

The aim of this experiment was to (1a) determine how exposure to recurring environmental disturbances of a specific type (here temperature pulses of different intensities) affects the resistance and recovery of a microbial community and (1b) how this is affected by disturbance intensity. Furthermore, we aimed to (2) test how disturbance history affects the resistance and recovery in response to a novel disturbance. Specifically, we hypothesized that resistance and recovery of bacterial communities would show a cumulative, directional change over time when communities are exposed to recurring disturbances, with a stronger response to stronger disturbances. We further hypothesized that recurring exposure to disturbances of one type (temperature) would affect the resistance and recovery of the community in response to a second, new disturbance (acidification).

### Experiment 1: Effect of repeated temperature disturbances at different intensities

Bacterial community composition changed in response to the disturbances and most of the changes in community profile already occurred in response to the first temperature pulse, in particular in the 35 °C treatment. It is therefore likely that the first pulse disturbance selected for the community members that could cope with the disturbance most efficiently. In addition, our results indicate a larger change in community composition for the 35 °C than for the 25 °C treatment. This is in agreement with results from previous studies using other types of disturbances, which showed, for example, that bacterial community composition changed gradually with increasing intensities and frequencies of a salinity disturbance^13,32^ or increasing concentration of a pollutant^14^. Moreover, the results clearly show a lack of compositional recovery in response to the applied temperature disturbances.

Another aspect of community structure that can be affected by disturbances is alpha diversity, including both richness and evenness. Generally, exposure to a disturbance may lead to a decrease in diversity^33,34^. In our study we found that richness decreased in all treatments, including the controls, between day 0 and 7 indicating the presence of an initial lab effect. In the 35 °C treatment richness continued to decrease after the second and third disturbances, whereas evenness remained constant after the initial decrease. Hence, our results show that repeated disturbances of high intensity modify in particular the richness of bacterial communities. Soil bacterial diversity has previously been shown to decline with increasing disturbance frequency^35^. Here we show that the intensity of the disturbance appears to be important too, since the second to fourth disturbance only caused an additional effect on richness in the 35 °C treatment, but not in the 25 °C treatment. This is consistent with another study in aquatic communities, showing that the interaction between disturbance frequency and intensity determines the diversity of the bacterial community^36^. It is therefore hard to predict how diversity will be affected by disturbances, but from our study it is clear that the 35 °C temperature disturbances were intense enough to cause a loss of species in the community, even after several previous exposures.

Changes in community composition that occur in response to disturbances can also affect community function and functional recovery and resilience. In previous experiments, communities exposed to disturbances have shown enhanced physiological tolerance and substrate utilization, which suggests proliferation and dominance of generalist bacterial populations^30,31^. This is in agreement with findings that generalist species are commonly associated with disturbed and heterogeneous environments, and specialist species with stable and homogeneous habitats^37,38^. One way of estimating enhanced physiological tolerance is through niche width. Here we found that presence-absence based niche width (which is a measure for the mean level of generalization of populations within the community^39^) initially increased in all treatments, suggesting a bottle effect, but subsequently decreased again over time in the 35 °C treatment to reach the same level as on day 0. Abundance-weighted niche width showed a similar pattern, but in the 35 °C treatment it started decreasing immediately. This indicates that the communities in the 35 °C treatment shifted toward being proportionally more dominated by specialist species, and this shift was noticeable as a change in abundance before the change in species presence. It seems therefore that niche width depended on both the frequency and intensity of the disturbance, with generalists being selected at low and specialists at high intensities. The 35 °C temperature disturbances seemed to be beyond the tolerance limits of the generalists in our experimental community, leading to a gradual selection for a more specialist community in that treatment. This is in line with what is seen in cases of stress-induced community tolerance^29,40^.

On the other hand, the functional response did not provide clear evidence of increased community process rates after exposure to the disturbances, as would be expected from the community tolerance framework^29,40^. Although bacterial abundance and bacterial carbon production increased in the control and 25 °C treatment from the second disturbance onward, bacterial carbon production stayed behind in the 35 °C treatment and abundance showed a similar trend, though to a lesser degree. In contrast, there was an opposite pattern in the extracellular enzyme activities. So, despite the unexpected but clear selection for specialists in the 35 °C treatment, this did not result in a unidirectional change in community process rates, but rather, the effect depended on the process that was measured, as well as the number of disturbances involved. This is in line with previous studies showing the importance of disturbance intensity^13,41^ and the function measured^13,22,42^ for the functional response of microbial communities. One explanation for the latter is that the degree of response to a disturbance might depend on the specificity of the function measured^43^. This fits with our observation of the strongest responses in the enzyme activities, which can be seen as more specific functions than bacterial carbon production and abundance.

Finally, our results confirm the apparent contradiction found in the literature, based on which disturbances are both thought to increase^29,40,41^ and decrease^22,24^ resistance and resilience to future disturbances. For both bacterial abundance and production, we found a slight negative effect of the disturbances on the response ratios in the 35 °C treatment, indicating a reduction in resistance and recovery after several disturbances. For the enzyme activities, however, we found that the response ratios gradually increased after each consecutive disturbance, despite a large decrease directly after the first exposure. Overall, the results of our experiment therefore suggest that exposure to repeated disturbances of the same type can gradually increase the functional resistance and recovery of the microbial community but that this depends on the strength of the disturbance and functional parameter measured.

### Experiment 2: Resistance to acidification in response to disturbance history

The pH disturbance did not have a strong effect on the taxonomic composition, although the history of temperature disturbances affected different functional parameters in different ways in response to the acidification pulse. For bacterial carbon production response ratios were significantly higher in the 35 °C treatment than in the other two treatments, which indicates that the repeated strong temperature disturbances led to increased resistance to and/or recovery from the pH disturbance. This is contrary to earlier studies on soil microorganisms, where previous exposure to a disturbance had a destabilizing effect on the response to new disturbances^22,24^, probably due to the need for stressed cells to allocate energy to, for example, detoxification and damage repair after the first disturbance, making additional disturbances harder to cope with^44,45^. In our case, however, bacterial carbon production and enzyme activity in the 35 °C treatment even exceeded the control values after the acidification event, similar to other studies that have shown that disturbances might enhance community function^13,46,47^. This confirms that prior disturbances can increase community tolerance and make the system more resistant to additional stressors^19,48^. This can be explained by acquired stress resistance, where cellular responses to a first disturbance can lead to lower metabolic costs when exposed to a new disturbance, impacting community composition through changes in survival and succession^26^. In addition, fluctuating environments^38,49,50^ and disturbances are believed to select for generalists^30,31^. Generalists have broader tolerance to environmental conditions, which could explain increased resistance and recovery of bacterial communities with larger proportions of generalists^30,31^. However, in our study average niche width as well as richness and evenness, were significantly lower in the 35 °C treatment directly after the acidification event (day 28), than in the other treatments. Together, this suggests that specialists rather than generalists were selected and that specific phylotypes became important for the response to the acidification event.

A straightforward explanation for the observed results would be that the response mechanisms related to the disturbances are similar. Selection pressure for one stress response mechanism would then automatically favour organisms with a higher tolerance to the other stressor. Most research on stress responses has been done in lactic acid bacteria and heat shock responses are described to include high production of heat shock proteins which are important in regulation of cellular repair processes such as refolding of damaged proteins^51^. Acid stress, on the other hand, induces a number of general shock responses, such as production of shock proteins (e.g. heat-shock proteins) and chaperones^51,52^. In addition, responses specific to acid stress include mechanisms for proton removal, production of substances to increase the pH in the cell and changes in cell-wall composition^52^. The physiological response to heat exposure may only be of partial advantage in the case of exposure to low pH. This might explain why we did not see a positive response for all functional parameters. However, it has also been shown that temperature stress as the priming stress category is effective in cross protection and can probably be explained by temperature stress inducing pathways related to general stress responses which affect the cell membrane and wall, folding of proteins and trehalose biosynthesis pathways^27^. In our experiment, the 25 °C treatment was likely too mild to activate the heat shock response, which could explain the differences between the 35 °C treatment and the other two treatments. The most probable explanation for higher tolerance to the pH disturbance in communities with the strongest temperature disturbance history could therefore be a combination of selection of specific phylotypes and acquired stress resistance. To conclude, our results suggest that the effect of disturbance history on the resistance of a microbial community to other kinds of disturbance likely depends on the specific mechanism of selection that the disturbance history had on the community^22^. If a certain type of disturbance selects for organisms with a specific set of response mechanisms which are also helpful to deal with the second type of disturbance, resistance and resilience will increase, whereas it will decrease or have a neutral effect when that is not the case^22^. Thus there might be no clear general patterns in how disturbance history affects resistance and resilience of microbial communities.

## Conclusions

Our experiment shows that repeated disturbances of a single type can lead to a gradual increase in functional resistance and recovery in a bacterioplankton community, and that this disturbance history may also lead to increased functional resistance and recovery in response to a novel disturbance.

However, our results also show that the community response (functional or compositional) is dependent on the parameter that is measured, and that the effect of a history of one type of disturbance on the response to a novel disturbance likely depends on the similarity of the stress responses and selection mechanisms.

Overall, this means that future studies need to move beyond the search for patterns in disturbance history effects on community composition and functioning in general, and rather focus on the mechanisms behind community level stress responses and selection pressures, and how these lead to functional and compositional stability.

## Methods

### Experimental set-up

**Figure 7:**
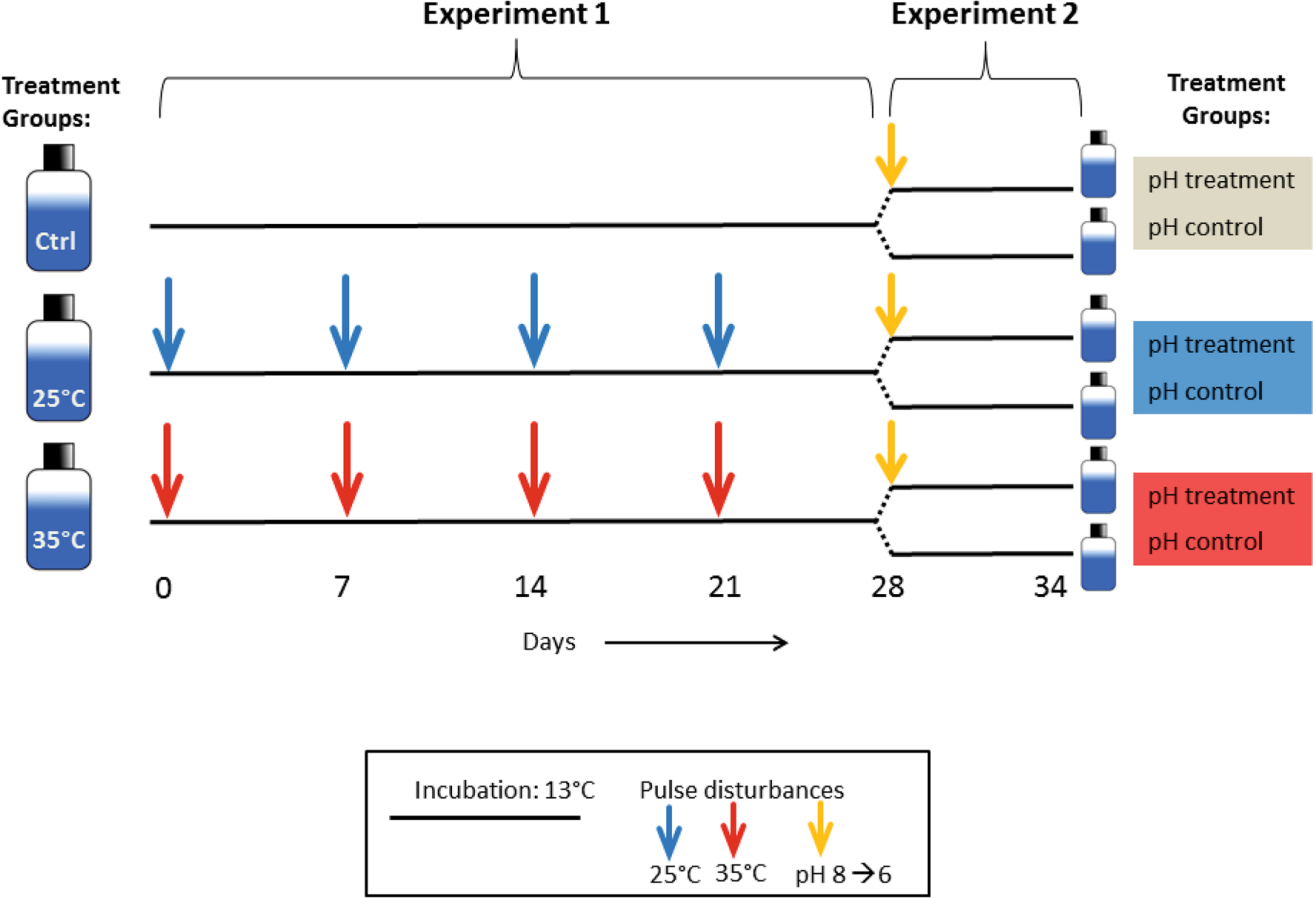
Overview of the experimental set-up. In Experiment 1 bacterioplankton communities were exposed to weekly temperature pulse disturbances of either 25 °C or 35 °C, while controls (ctrl) were kept at the incubation temperature of 13 °C (4 replicates each). In total, 4 pulses were applied over 4 weeks: on days 0, 7, 14 and 21. For Experiment 2, each culture was split in half on day 28, and one half received an acid pulse, temporarily reducing the pH from 8 to 6, while the other (pH control) remained undisturbed for an additional 6 days. All incubations took place at 13 °C.

A microcosm experiment was set up where a natural lake bacterial community was subjected to a series of pulse disturbances over a 5-week period. Two types of disturbances were chosen; changes in temperature and pH (Fig. 7).

In Experiment 1, the communities were exposed to temperature pulse disturbances of two different intensities for 15 h every 7th day for a period of 4 weeks. The length of the disturbances was based on the time needed to heat the bacterial communities in their growth medium to the desired temperature, while avoiding exposure of multiple bacterial generations to the disturbance. The 7-day disturbance interval was chosen to have the potential for recovery of community functioning between disturbances, based on the slow recovery rates observed in pre-tests with a single disturbance. While one treatment group remained undisturbed and was kept at 13 °C (Control), the two other treatment groups were heated to 25 °C (25 °C treatment) or 35 °C (35 °C treatment) in an incubation chamber, before being returned to 13 °C (Fig. 7). Each treatment was applied to four replicate communities. The incubation temperature of 13 °C was chosen to maximize bacterial activity while still being suitable for cold-adapted species^53^, as we collected our samples during autumn. The disturbance levels of 25 °C and 35 °C were chosen because they represent the highest yearly water temperature at the sampling site^54^ and a temperature that might potentially occur under extreme summer heat events.

In Experiment 2, we then investigated how the temperature disturbance history affected the resistance to another type of disturbance. One week after the last temperature disturbance (day 28), the volume of each replicate microcosm was split into two equal parts, with one exposed to an additional acidification event and the other remaining as a control. The reduction by two pH units (from pH 8 to pH 6 by adding hydrochloric acid) was used to introduce a strong disturbance and was chosen based on previous findings that community tolerance for pH generally is ± 1 pH unit^55^. The incubation was then continued for six more days (until day 34, Fig. 7). Before splitting the mesocosms, one replicate from the 35 °C treatment was accidentally lost. As a result, only three replicates remained in both the pH treatment and the control group for the 35 °C treatment groups in Experiment 2 (Fig. 7).

### Sampling and preparation of medium and inoculum

On October 27th 2014 eighty litres of water were collected from the Ekoln basin in Lake Mälaren, Sweden (59°45′48.99″N, 17°34′33.09″E). The water was transported back to the laboratory within one hour and stored at 4 °C until processing.

The growth medium was prepared by sterile-filtering the water through 0.2 µm membrane filters (Pall corporation), followed by autoclaving. The medium was then stored at 4 °C until use and autoclaved again just before use. This procedure caused a pH change from pH 7.8 to pH 8.8, which was compensated with hydrochloric acid.

For the inoculum (initial community), 20 L of water was collected on November 10th, 2014 from the same location (8.2 °C in situ temperature), filtered through a GF/F glass microfiber filter (0.7 µm, Whatman) to remove bacterial grazers and stored at 13 °C to acclimatize the bacterial communities for 2 days before starting the experiment. A daily 20% medium exchange was performed to avoid nutrient depletion. The culture medium removed during this process was used to measure community parameters.

### Community composition

Samples for the community composition analysis were taken just prior to each disturbance and six days after the pH disturbance (day 0, 7, 14, 21, 28 and 34). Bacterioplankton cells were collected by filtering 100 mL of culture onto 0.2 µm membrane filters (Pall Corporation). Filters were stored at − 80 °C. DNA was extracted using the Power Soil DNA isolation kit (Mo BIO laboratories, Carlsbad, Ca, USA) and quantified with the Quant-iT PicoGreen dsDNA Assay Kit (Life Technologies, USA). Extracted DNA was stored at − 20 °C. The bacterial 16S rRNA gene was first amplified using bacterial primers 341F and 805R (V3 and V4 of the ribosomal gene) containing an adaptor. Amplification was performed using the following PCR conditions; 98 °C initial denaturation for 30 s, followed by 20 cycles of 98 °C for 10 s, 62 °C for 30 s, and 72 °C for 30 s, and a final extension at 72 °C for 2 min.

Individual samples were then labelled with barcodes following the protocol by Sinclair, et al.^56^. The resulting barcoded amplicons were purified using magnetic beads (Agencourt AMpure XP) and then normalized in equimolar amounts and sequenced on a MiSeq system at the SciLifeLab, Uppsala, Sweden. Raw sequence data were processed using the UPARSE pipeline^57^ and taxonomically identified using the SINA/SILVA database. Sequences from all treatments were clustered together into operational taxonomic units (OTU) using Usearch^58^. After quality control our data consisted of 31 368 ± 17 662 reads per sample and the final OTU table resulted in 510 OTUs (excluding singletons) delineated at 97% 16S rRNA gene identity. DNA sequences have been deposited in the National Center for Biotechnology Information (NCBI) Sequence Read Archive under accession number PRJNA534401.

The high annealing temperature in the first PCR step might have resulted in an underestimation of Alpha-Proteobacteria as certain groups with mismatches to the 805R primer have been shown not to amplify under such conditions^59^. Therefore, we compared the general patterns of community composition captured by the sequence data with a TRFLP analysis based on less stringent PCR conditions that was performed on the same samples (see Supplementary Methods for details on the TRFLP preparation and results). Overall, highly similar results were found and we therefore conclude that the sequence analysis data is suitable for the comparative community analysis in this study.

Subsampling was performed to 5,000 reads per sample and samples with lower numbers of sequences were removed. Richness (S.Obs, observed number of species) and evenness were calculated in R 3.0.2 using the package vegan^60^.

To investigate how the proportion of generalists changed during the experiment, habitat specialization was calculated for each sample at each time point using Levins’ niche width (B) index^39^ (see Supplementary Methods).

### Community functioning

Samples for total bacterial abundance (as a proxy for biomass) were taken on a daily basis and preserved by adding formaldehyde to a final concentration of 2%. Cells were stained with SYTO 13 solution (1.25 μM, Molecular Probes)^61^, and their abundance was determined using a Cyflow flow cytometer (Partec, Münster, Germany).

Bacterial carbon production was measured every day using leucine incorporation^62^. L-[4, 5-3*H*] Leucine (Perkin Elmer) was diluted to 15% with unlabelled l-Leucine (Sigma, St Louis, MO, USA) and added at a final concentration of 100 nM. Samples and blanks were incubated at 13 °C for 1 h. Disintegrations per minute (DPM) was recalculated to bacterial carbon production rates (ng C L^−1^ h^−1^)^63^.

The activities of cellobiohydrolase and β-glucosidase were measured prior to each disturbance and six days after the pH pulse disturbance. Enzymatic activities were measured using methylumbelliferone (MUF)-linked substrates (Sigma-Aldrich) under saturating conditions (0.6 mM final conc.). The samples, blanks and MUF standards were incubated for 3 h in the dark at room temperature. After incubation, glycine buffer (pH 10.4) was added (1:1 v:v) and fluorescence was measured at λ_ex/em_ = 360/465 nm (Ultra 384, Tecan, Switzerland)^64^.

### Statistical analysis

The effects of treatment on bacterial abundance, bacterial carbon production, β-glucosidase activity and cellobiohydrolase activity (Experiment 1) were analysed by multivariate repeated-measures ANOVA (rm ANOVA; using the JMP 11 statistical software). Time was analysed as a fixed factor, rather than a continuous covariate, to account for non-monotonic changes over time. As a result, each day was fitted as a separate variable^65^. To avoid loss of replication, days 3, 4 and 6 were excluded from the bacterial abundance measurement, and days 4, 8 and 23 were removed from the production measurements. The multivariate rm ANOVA gives similar outcomes to the linear mixed model approach, provided the assumption of sphericity is met^65^. In cases where the assumption of sphericity was violated, the Greenhouse–Geisser (G–G) correction was applied.

To determine functional resistance and recovery, response ratios were calculated as the proportion of a functional variable measured in the disturbed treatment to the control treatment at the same time^7^. Response ratios were used as a measurement of resistance directly after the disturbance as well as recovery over time. In Experiment 1 response ratios were calculated for bacterial abundance, bacterial carbon production, β-glucosidase activity and cellobiohydrolase activity as the ratio of the mean values in the different treatments and those of the control.

In Experiment 2 response ratios for bacterial abundance and bacterial carbon production with respect to the pH disturbance were calculated for each temperature treatment and subsequently differences in response ratios between the different temperature disturbance histories were tested using rm ANOVA (as described above). In addition, differences in response ratios for bacterial abundance, bacterial carbon production and extracellular enzyme activities between temperature treatments at each time point were analysed using separate one-way ANOVA, followed by a Tukeys HSD (R statistical software). Changes in community composition were visualized with non-metric multidimensional scaling (NMDS) ordination plots using Bray–Curtis dissimilarities of the sequence data (R statistical software, Vegan package). Average Bray Curtis dissimilarities were calculated between each time point and for each treatment to quantify differences. Richness, evenness, niche width and weighted niche width results were analysed using general linear mixed model ANOVA (mm ANOVA; JMP). Microcosm ID was modelled as a random factor. Differences between treatments at the various time points were tested using Tukey’s HSD (JMP).

## Supplementary information

Supplementary file1

## Data Availability

DNA sequences have been deposited in the National Center for Biotechnology Information (NCBI) Sequence Read Archive under accession number PRJNA534401. The functional datasets generated and analysed during the current study are available in the DiVA repository: https://urn.kb.se/resolve?urn=urn:nbn:se:uu:diva-409572.
