# Supplementary material for: Disturbance history can increase functional stability in the face of both repeated disturbances of the same type and novel disturbances
Source: Sci Rep. 2020 Jul 9;10:11333. doi: 10.1038/s41598-020-68104-0 (PMC7347917; doi:10.1038/s41598-020-68104-0)
Supplement: Supplementary file 1 — Supplementary file1 [file 41598_2020_68104_MOESM1_ESM.pdf]

Disturbance history can increase functional stability in the face of both repeated disturbances of the same type and novel disturbances

Sophia Elise Renes, Johanna Sjöstedt, Ingo Fetzer, Silke Langenheder

Supplementary information

Tables

Table S1. Average Bray Curtis dissimilarity between different days and treatments. Number within parentheses represents standard deviation.

**Treatment**	**Exp 1 Day 0 to 7**	**Exp 1 Day 7 to 14**	**Exp 1 Day 14 to 21**	**Exp 1 Day 21 to 28**	**Exp 2 Day 28 to 34 Control**	**Exp 2 Day 28 to 34 Shock**
**Control**	0.578 (0.0658)	0.347 (0.159)	0.375 (0.142)	0.391 (0.148)	0.384 (0.0981)	0.366 (0.115)
**25°C**	0.528 (0.0377)	0.280 (0.0447)	0.473 (0.108)	0.401 (0.0732)	0.317 (0.110)	0.392 (0.0627)
**35°C**	0.910 (0.0156)	0.388 (0.161)	0.368 (0.0851)	0.344 (0.144)	0.434 (0.0780)	0.548 (0.135)

Table S2. Results from general linear mixed model ANOVAs testing the effects of repeated temperature pulse disturbances and time on richness, evenness, niche width and weighted niche width in Experiment 1. Degrees of freedom (DF) in the denominator are presented in italics, DF in the numerator are presented in regular font.

	Richness			Evenness		
Factor	DF	F	P	DF	F	P
Between-subject effects	*9.3*			*8.3*		
temperature pulse	2	9.783652	**0.0052**	2	26.68641	**<0.0002**
Within-subject effects	*27*			*27.2/27.1**		
Time	4	44.75201	**<0.0001**	4	14.80338	**<0.0001**
Time x temperature pulse	8	3.147241	**0.0120**	8	3.182430	**0.0112**
	Niche Width			Weighted Niche Width		
Factor	DF	F	P	DF	F	P
Between-subject effects	*8.8*			*5.9*		
temperature pulse	2	17.08350	**0.0009**	2	216.6962	**<0.0001**
Within-subject effects	*25.5*			*25.7/25.6**		
Time	4	75.77726	**<0.0001**	4	14.60281	**<0.0001**
Time x temperature pulse	8	12.19388	**<0.0001**	8	18.91690	**<0.0001**

* The second value is for the interaction term

Table S3. Tukey's HSD results of pairwise comparisons of richness, evenness, niche width and weighted niche width in Experiment 1. Only the comparisons within days are presented. Numbers presented are p-values.

Test -Richness	Day 0	Day 7	Day 14	Day 21	Day 28
Control - 25	0.9992	0.9998	0.8937	1.0000	1.0000
Control - 35	1.0000	1.0000	0.9965	**0.0236**	**0.0143**
25 - 35	0.9990	0.9755	0.3754	**0.0070**	**0.0293**
Evenness					
Control - 25	1.0000	0.9999	1.0000	0.9544	1.0000
Control - 35	1.0000	0.1470	0.0848	**0.0050**	**0.0286**
25 - 35	1.0000	**0.0258**	0.0779	**0.0001**	**0.0125**
Niche Width					
Control - 25	1.0000	0.9990	0.9963	0.9906	1.0000
Control - 35	1.0000	**0.0375**	**0.0139**	**<0.0001**	**<0.0001**
25 - 35	1.0000	0.2844	0.3208	**0.0004**	**<0.0001**
Weighted Niche Width					
Control - 25	0.9744	1.0000	0.9802	0.9849	0.8754
Control - 35	1.0000	**<0.0001**	**<0.0001**	**<0.0001**	**<0.0001**
25 - 35	0.9965	**<0.0001**	**<0.0001**	**<0.0001**	**<0.0001**

Table S4. Results from repeated-measures ANOVAs testing the effects of repeated temperature pulse disturbances and time on abundance (BA), bacterial production (BCP), β-glucosidase activity (BG) and cellobiohydrolase activity (CBH) in Experiment 1. The p-values are Greenhouse Geisser adjusted. Degrees of freedom (DF) in the denominator are presented in italics, DF in the numerator are presented in regular font.

	BA			BCP		
Factor	DF	F	P	DF	F	P
Between-subject effects	*9*			*9*		
temperature pulse	2	6.0461	**0.0217**	2	0.4426	0.6556
Within-subject effects	*35.527*			*27.902*		
Time	3.9474	52.52	**<0.0001**	3.1002	34.4414	**<0.0001**
Time x temperature pulse	7.8948	4.4427	**0.0009**	6.2005	3.6787	**0.0076**
	BG			CBH		
Factor	DF	F	P	DF	F	P
Between-subject effects	*9*			*9*		
temperature pulse	2	17.6541	**0.0008**	2	5.4491	**0.0281**
Within-subject effects	*14.351*			*15.787*		
Time	1.5945	109.97	**<0.0001**	1.7542	52.6606	**<0.0001**
Time x temperature pulse	3.189	11.5650	**0.0004**	3.5083	6.07	**0.0046**

Table S5. Results from repeated-measures ANOVAs testing effect of temperature disturbance history and time on response ratios of bacterial cell abundance (BA) and bacterial carbon production (BCP) calculated between pH disturbance and control treatments in Experiment 2. The p-values are Greenhouse Geisser adjusted. Degrees of freedom (DF) in the denominator are presented in italics, DF in the numerator are presented in regular font.

	BA			BCP		
Factor	DF	F	P	DF	F	P
Within-subject effects	*15.293*			*17.756*		
Time	1.9116	0.414	0.6591	2.5366	0.268	0.8162
Time x temperature history	3.8233	0.152	0.9551	5.0732	0.7809	0.5781
Between-subject effects	*8*			*7*		
Temperature history	2	9.246	**0.0083**	2	10.03	**0.0088**

Table S6: Results from separate ANOVAs for each time point in Experiment 2 to investigate the effect of treatment on the response ratios based on bacterial abundance (BA), bacterial carbon production (BCP), cellobiohydrolase activity (CBH) and β-glucosidase activity (BG).

		Day 29		Day 30		Day 32		Day 34	
Index	Factor	$F_{2,8}$	P	$F_{2,8}$	P	$F_{2,8}$	P	$F_{2,8}$	P
BA	Treatment	1.551	0.27	0.562	0.591	1.019	0.403	0.716	0.517
Index	Factor	$F_{2,8}$	P	$F_{2,7}$	P	$F_{2,8}$	P	$F_{2,8}$	P
BCP	Treatment	1.012	0.406	2.189	0.183	2.106	0.184	14.23	**0.0023**
CBH	Factor							$F_{2,8}$	P
	Treatment							3.831	0.0681
BG	Factor							$F_{2,8}$	P
	Treatment							2.886	0.114

Methods

Community composition determined by terminal restriction fragment length polymorphism (T-RFLP)

Samples for the community composition analysis were taken just prior to each disturbance and six days after the pH disturbance. Community profiles were constructed from samples taken at day 0, 14, 21, 28 and 34 using terminal restriction fragment length polymorphisms (T-RFLP) [1]. Bacterioplankton cells were collected by filtering 100 mL of culture onto 0.2 µm membrane filters (Pall Corporation). Filters were stored at -80°C. DNA was extracted using the Power Soil DNA isolation kit (Mo BIO laboratories, Carlsbad, Ca, USA) and quantified with the Quant-iT PicoGreen dsDNA Assay Kit (Life Technologies, USA). Extracted DNA was stored at -20°C. PCR of the 16S rRNA gene was performed using the EUB-8-forward primer, labelled with hexachlorofluorescein (HEX) (5-AGRGTTTGATCMTGGCTCAG-3; Thermo Scientific, USA) and the reverse primer 519R (5-GWATTACCGCGGCKGCTG-3; Invitrogen, USA). The 50 µL PCR reaction contained, up to 10 ng of DNA extract, 1xNH_4 buffer (Bioline), 1.5 mM of $MgCl_2$ (Bioline), 250 µM of dNTPs (Thermo Scientific), 200 nM of each primer and 0.05 U/µL BioTaq DNA polymerase (Bioline). PCR was performed using the following conditions; initial denaturation for 3 minutes at 94°C; 25 cycles of 94°C for 30 sec, 52°C for 30 sec, 72°C for 45 sec; and a final extension at 72°C for 7 min.

DNA was purified using QIAquick PCR purification kit (Qiagen, Germany), and eluted in 30 µL elution buffer to increase the concentration. Concentrations were determined with the Quant-iT PicoGreen dsDNA Assay Kit (Life Technologies, USA). The restriction digestion was performed according to a modified Terminal restriction fragment length polymorphism (T-RFLP) protocol [2]. Approximately 30-50 ng DNA from each sample was restricted (in duplicate) using 4 U HaeIII restriction enzyme per reaction in 1x Buffer Components (New England Biolabs, USA), with a total restriction volume of 10 µL. The samples were incubated for 18 hours at 37°C in the dark. HEX-labelled terminal restriction fragments were then separated on an ABI 3700 96-capillary sequencer running in GeneScan mode (Applied Biosystems) together with an internal size standard at the SciLife Laboratory (Uppsala, Sweden).

Community profiles were established using SoftGenetics GeneMarker (2.6.4) software. For all fragments (50 and 500 nucleotides) the fluorescence intensities (calculated from the heights of the peaks) were reported in a binning table. Signals that were only present in one replicate from a sample were removed, after which mean values of the relative fluorescence intensities were

calculated from the two replicates. All fragments with a relative intensity <0.5% of total signal intensity were excluded.

Calculation of habitat specialization

Habitat specialization was calculated for each sample at each time point using Levins' niche width (B) index, as shown in equation (1)[3].

$$B = 1/\sum_{i=1}^{N} p_{ij}^2 \qquad (1)$$

where p_{ij} is the proportion of OTU j in the sample i at a specific time, and N is the number of samples. Average niche width was calculated in two different ways, with and without taking the abundance of each OTU present in each sample into account (abundance-weighted and presence-absence). Using these two indices together makes it possible to disentangle changes in species proportions (abundance-weighted) from species extinction (presence-absence).
In both cases higher niche width indicates higher dominance of generalist in the communities.

References

1 Liu, W. T., Marsh, T. L., Cheng, H. & Forney, L. J. Characterization of microbial diversity by determining terminal restriction fragment length polymorphisms of genes encoding 16S rRNA. *Applied and Environmental Microbiology* **63**, 4516-4522 (1997).

2 Logue, J. B. & Lindstrom, E. S. Species sorting affects bacterioplankton community composition as determined by 16S rDNA and 16S rRNA fingerprints. *Isme J.* **4**, 729-738, doi:10.1038/ismej.2009.156 (2010).

3 Pandit, S. N., Kolasa, J. & Cottenie, K. Contrasts between habitat generalists and specialists: an empirical extension to the basic metacommunity framework. *Ecology* **90**, 2253-2262, doi:10.1890/08-0851.1 (2009).

Figures

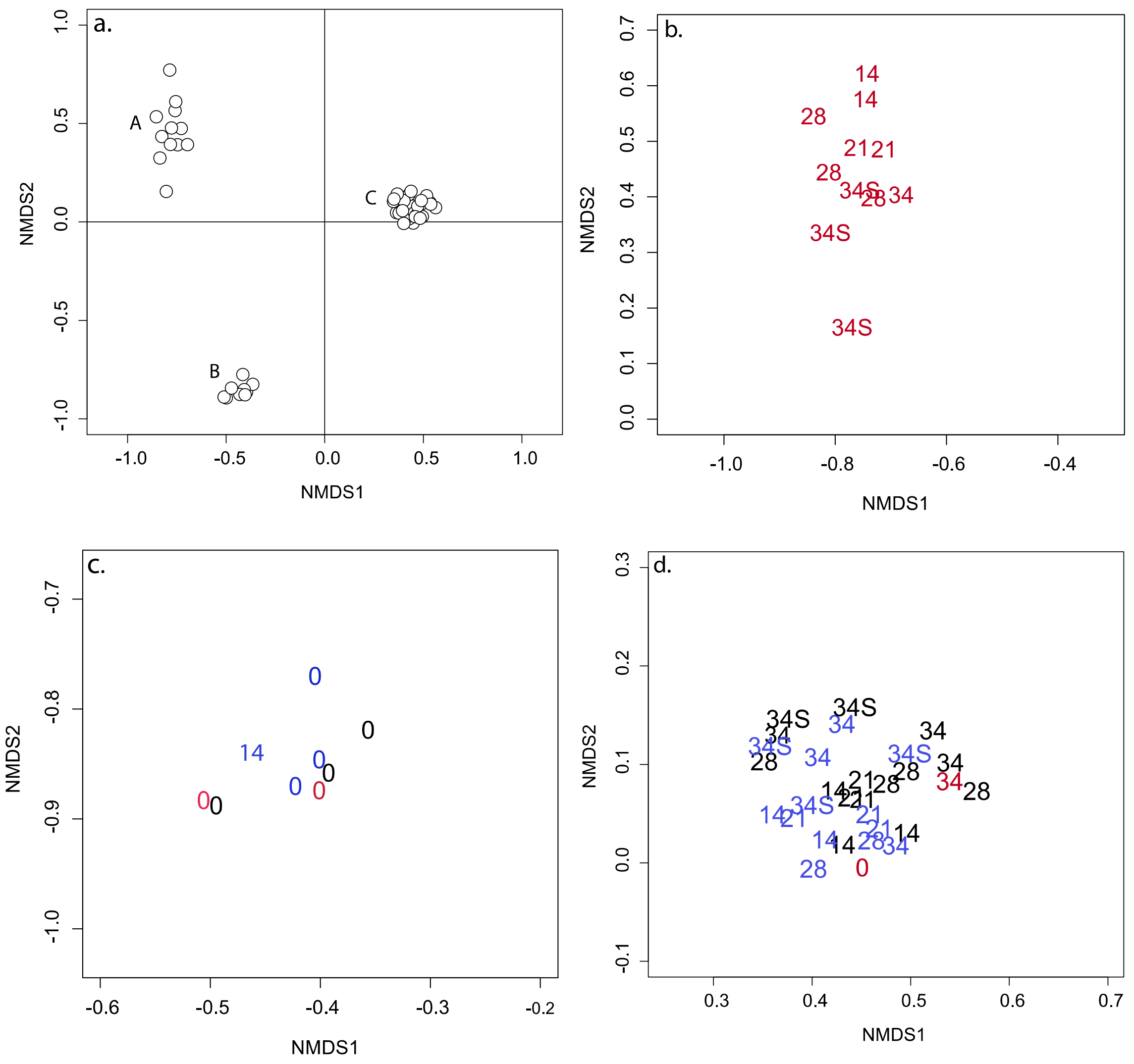

Figure S1. **NMDS ordination plot showing changes in bacterial community composition over time.** Analysis is based on TRFLP data and Bray-Curtis dissimilarities. A) overall ordination. Samples were separated into three clusters, Cluster A, Cluster B and Cluster C. B-D are zoomed in on the three clusters to show more detail. B) Cluster A, C) Cluster B and C) Cluster C. Treatment groups are indicated by the colour (Black = Control, Blue = 25°C treatment, Red = 35°C treatment). In Experiment 2 the cultures were split in half and one half was subjected to an acid shock while the other half served as controls. At day 34 S indicates that the culture has been exposed to acid shock. The three clusters showed separation based on sampling day and treatment. Cluster 'A' (Fig. S1), exclusively contained samples from the 35°C treatment later than day 0. Cluster 'B' contained all except 1 of the samples from day 0 (35°C treatment) and one sample from day 14 (25°C treatment). Finally, Cluster 'C' contained all the samples from the control and 25°C treatment after day 0, as well as one day 0 and one day 34 sample from the 35°C treatment. Stress: 0.05.
